# A Graph Theory Approach to Clarifying Aging and Disease Related Changes in Cognitive Networks

**DOI:** 10.3389/fnagi.2021.676618

**Published:** 2021-07-12

**Authors:** Laura M. Wright, Matteo De Marco, Annalena Venneri

**Affiliations:** ^1^Department of Neuroscience, University of Sheffield, Sheffield, United Kingdom; ^2^Department of Life Sciences, Brunel University London, London, United Kingdom

**Keywords:** Alzheimer’s disease, network analysis, mild cognitive impairment, aging, cognition

## Abstract

In accordance with the physiological networks that underlie it, human cognition is characterized by both the segregation and interdependence of a number of cognitive domains. Cognition itself, therefore, can be conceptualized as a network of functions. A network approach to cognition has previously revealed topological differences in cognitive profiles between healthy and disease populations. The present study, therefore, used graph theory to determine variation in cognitive profiles across healthy aging and cognitive impairment. A comprehensive neuropsychological test battery was administered to 415 participants. This included three groups of healthy adults aged 18–39 (*n* = 75), 40–64 (*n* = 75), and 65 and over (*n* = 70) and three patient groups with either amnestic (*n* = 75) or non-amnestic (*n* = 60) mild cognitive impairment or Alzheimer’s type dementia (*n* = 60). For each group, cognitive networks were created reflective of test-to-test covariance, in which nodes represented cognitive tests and edges reflected statistical inter-nodal significance (*p* < 0.05). Network metrics were derived using the Brain Connectivity Toolbox. Network-wide clustering, local efficiency and global efficiency of nodes showed linear differences across the stages of aging, being significantly higher among older adults when compared with younger groups. Among patients, these metrics were significantly higher again when compared with healthy older controls. Conversely, average betweenness centralities were highest in middle-aged participants and lower among older adults and patients. In particular, compared with controls, patients demonstrated a distinct lack of centrality in the domains of semantic processing and abstract reasoning. Network composition in the amnestic mild cognitive impairment group was similar to the network of Alzheimer’s dementia patients. Using graph theoretical methods, this study demonstrates that the composition of cognitive networks may be measurably altered by the aging process and differentially impacted by pathological cognitive impairment. Network alterations characteristic of Alzheimer’s disease in particular may occur early and be distinct from alterations associated with differing types of cognitive impairment. A shift in centrality between domains may be particularly relevant in identifying cognitive profiles indicative of underlying disease. Such techniques may contribute to the future development of more sophisticated diagnostic tools for neurodegenerative disease.

## Introduction

The integrity of our cognitive functions is heavily influenced by a number of factors including, but not limited to, our educational background (Katzman, 1993), age (Glisky, 2007; Harada et al., 2013), and the structure and function of the physiological systems that underlie them. Although more heavily exacerbated in disease, declines in cognitive function are also inherent to healthy aging (Salthouse, 2003; Harada et al., 2013). As aging itself is a major risk factor associated with the development of dementia due to multiple etiologies, it is imperative that clear distinctions may be drawn between what can be determined age-related and pathology-related cognitive decline.

Despite recent advances in biomarker identification (Olsson et al., 2016; Jack et al., 2018), clinical diagnosis of many neurodegenerative diseases continues to rely on the detection of distinct cognitive or behavioral changes, characteristic of a given disease (National Institute for Health and Care Excellence, 2018). Due to the heterogeneous nature of neurodegenerative conditions, particularly in the prodromal stages (Petersen, 2004; Ismail et al., 2016), evaluations of individual cognitive functions in this manner tend to be limited in their ability to differentiate accurately between etiologies and predict future progression to dementia (Loewenstein et al., 2006; Fischer et al., 2007).

Although the traditional reductionist approach toward the study of cognitive functioning is clinically helpful and provides a valuable theoretical avenue for the formulation of inter-disciplinary research hypotheses (Barendregt and van Rappard, 2004), a more global, non reductionist view is of help to characterize cognitive profiles in psychopathology in a way that is more attentive to the intertwined nature of symptoms (Borsboom et al., 2019). This would be particularly valuable in clinical neuropsychological practice, where diagnoses are formulated using profiles of test scores, resultant of inter-connected, rather than isolated functions.

In line with a non-reductionist view of cognition, one concept of senescent change, which may be captured via methods of network analysis, is that of cognitive dedifferentiation. Originally described by psychometric studies, cognitive dedifferentiation refers to the tendency for cognitive and sensory functions spanning differing domains to present with increased intra-individual correlation among aging populations (Baltes et al., 1980; Baltes and Lindenberger, 1997). Despite conflicting evidence in the literature regarding the existence of this phenomenon (de Frias et al., 2007; Tucker-Drob, 2009; La Fleur et al., 2018; Tucker-Drob et al., 2019), the *cognitive dedifferentiation hypothesis* remains a central concept within the study of aging and cognition, due largely to the assumption that the well-established dedifferentiation in neural function and response to cognitive tasks in older individuals (Koen and Rugg, 2019; Koen et al., 2020) is likely to result in greater correlations between subsequent performance on tasks of differing domains. Dedifferentiation, therefore, whether a factor of healthy aging or a manifestation of disease (Batterham et al., 2011), provides an example of network-level cognitive alteration that demonstrates how a non-reductionist approach to the global cognitive system may be beneficial to elucidate subtle changes beyond the level of individual abilities or behaviors.

Graph theory is a mathematical tool that allows topological quantification of any system that could reasonably be described as a network. In this case, a network comprises a set of entities, referred to as nodes, joined by a series of connections, referred to as edges (Bondy and Murty, 1976). Although adhering to distinct domains, cognitive functions do not exist in isolation from one another. Rather, successful performance of most tasks relies on the interdependence of a number of cognitive domains. The characteristic separation and integration of cognitive abilities, therefore, allows for the conceptualization of a cognitive network in which performance on each task corresponds to a node and the interrelatedness or correlation between performances, to an edge (Garcia-Ramos et al., 2016). Graph theoretical methods have rarely been applied to interrogate the nature of our cognitive systems. A number of studies by Garcia-Ramos et al. (2015, 2016) and Kellermann et al. (2015, 2016), probing the nature of cognition in epilepsy, have, however, exploited graph theory methods in this area to some effect, demonstrating the utility of the technique in identifying measurable differences in neuropsychological profiles between distinct groups. Only two previous studies have implemented similar network analyses comparing the cognitive profiles of healthy aging individuals to those with Alzheimer’s disease (AD). A recent study by Tosi et al. (2020) analyzed cognitive networks in healthy older adults, patients with AD and patients with vascular encephalopathy. Although this study demonstrated a number of qualitative differences in network characteristics between the healthy group and those with neurological disease, the results were limited in terms of quantitative differentiation between aging and disease-related cognitive network topology. Ferguson (2021) took the approach a step further, comparing the cognitive networks of healthy older adults to those with early Alzheimer’s type dementia and amnestic mild cognitive impairment, who may represent a prodromal stage of disease. This study was able to corroborate Tosi et al.’s findings, demonstrating substantial evidence for reorganization of the cognitive network in even the earliest stages of Alzheimer’s disease. What is lacking from the literature, however, is an exploration of cognitive network reorganization as it may occur throughout the stages of healthy aging, as conceptualized by the *cognitive dedifferentiation hypothesis*. Such establishment of a possible aging effect on network composition is important for identifying topological deviations that may be indicative of an age-related neuropathology. Furthermore, the assertion that pathological cognitive impairment may result in an abnormal reorganization of the cognitive network suggests that etiologies other than AD may similarly demonstrate such topological alterations. Given the varying cognitive profiles associated with differing disease etiologies, it is likely that the network topologies associated with other types of cognitive impairment will also differ, as seen in the comparison between patients with AD and patients with vascular encephalopathy in Tosi et al.’s (2020) study. The present study, therefore, applied methods of graph theory to evaluate differences in the structure of cognitive networks between healthy individuals of different age groups and in patients with different severities and sub-types of cognitive impairment, relating to neurodegenerative disease. Six network metrics that are frequently utilized by neuroimaging studies, assessing network integration, segregation, and modularity, were chosen to test the hypothesis that differences in network topology identified in Alzheimer’s disease at a neural level will similarly manifest in cognition. It was expected therefore, that the topology of cognitive networks highlighted by these metrics would show both quantitative and qualitative differences between the stages of healthy aging that would be distinct from network alterations associated with pathological cognitive decline.

Of particular interest to this study, was the hypothesis that as individuals age, crystallized cognitive abilities such as vocabulary, general knowledge, and semantic memory (Cattell, 1971) may be more heavily relied upon to support healthy cognitive function in the presence of age-related declines in domains such as processing speed, executive function, and episodic memory (Li et al., 2004; Harada et al., 2013). Throughout the lifespan, crystallized abilities have been shown to remain relatively stable, showing markedly low levels of decline in old age, compared with other cognitive domains (Cattell, 1971; Nyberg et al., 1996; Rönnlund et al., 2005), with some tests even being found to show gradual improvement between the decades of life, until around the age of 60 (Nilsson, 2003; Verhaeghen, 2003; Rönnlund et al., 2005; Salthouse, 2009, 2019). In contrast, however, patients with AD, even at a very early stage of disease, show declines in these areas, in particular in language and semantic memory function (Garrard et al., 2005; Amieva et al., 2008; Vonk et al., 2020). Through examination of the relationship between test performances in a range of cognitive domains, the present study aimed to test the hypothesis that compared with younger adults, older healthy adults will present with a cognitive network in which tests of semantic memory are highly influential. In patients with Alzheimer’s disease, however, even among those in a prodromal stage, the role of semantic processing in the cognitive network was expected to be significantly reduced, suggesting that even in the absence of a measurable decline in functioning, network analysis may reveal alterations in the inter-relatedness of cognitive domains, in particular their relation to crystallized abilities, that may serve to differentiate healthy aging from disease. As such, in line with the Alzheimer’s disease cognitive profile (McKhann et al., 2011), network differences in comparison with controls, among amnestic groups were expected to be most evident, in terms of the relationships between cognitive test performance, in the domains of semantic processing and memory function. Between the stages of healthy aging, however, it was expected that crystallized cognitive functions such as semantic processing (Cattell, 1971) may have a more prominent role in the network structure of older adults compared with younger groups, while substantial differences in network properties relating to tests of executive functioning may be further apparent, in line with age-related declines in this domain (Harada et al., 2013). Furthermore, in accordance with the findings of Ferguson (2021) and previous neuroimaging analyses (Rashidi-Ranjbar et al., 2020), patients with an amnestic mild cognitive impairment, and not those with a non-amnestic impairment, were expected to present with a network composition closely aligned to patients diagnosed with Alzheimer’s dementia.

## Materials and Methods

### Participants

The participant sample (*N* = 415 datasets) included in this study were identified retrospectively from a large database coordinated by the University of Sheffield’s Department of Neuroscience. Healthy adults (*n* = 220) were approached using multiple recruitment strategies, with a proportion being carers of patients and others obtained via opportunity sampling and assigned to one of three groups according to their age: a younger group aged 18–39 (*n* = 75), a middle-aged group aged 40–64 (*n* = 75) and an older group aged 65+ (*n* = 70). All patients were recruited through a memory clinic after neurological examination and were split according to clinical diagnosis. Of the 195 patients, 60 had a clinical diagnosis of probable Alzheimer’s disease dementia, in adherence to the NINCDS-ADRDA criteria (McKhann et al., 2011), and 135 received a diagnosis of mild cognitive impairment (MCI) who, following the criteria outlined in Albert et al. (2011), were further categorized into those with an amnestic MCI (aMCI) profile (*n* = 75) and those with a non-amnestic profile (naMCI) (*n* = 60) according to neuropsychological assessment. All procedures were carried out following the Declaration of Helsinki. This study received ethical approval from the West of Scotland Regional Ethics Committee 5, Ref. No.: 19/WS/0177. Written informed consent was obtained from all participants.

Demographic data for all participant groups can be found in Table 1. All patient groups were matched with the healthy older adults in terms of age and were further age and education matched with each other. All healthy groups were also education matched with each other. All patient groups, however, had significantly fewer years of education than each control group. There were no significant differences across any of the groups in terms of gender ratios.

**TABLE 1:** Median (and inter-quartile range) of demographics for participant groups.

	Young (*n* = 75)	Middle aged (*n* = 75)	Older (*n* = 70)	aMCI (*n* = 75)	naMCI (*n* = 60)	Dementia (*n* = 60)
Age	23.00 (10.00)^e^	53.00 (8.00)^b c^	72.00 (7.00)	75.00 (12.00)	71.00 (12.00)	74.50 (17.00)
Education	15.00 (3.00)	14.50 (5.00)	14.00 (4.00)	10.00 (5.00)^d^	12.00 (6.00)^d^	11.00 (5.00)^d^
Gender (M/F)	31/44	37/38	31/39	29/46	27/33	33/27
MMSE	29.00 (2.00)^a^	30.00 (1.00)	29.00 (2.00)^a^	26.00 (3.00)^d^	27.00 (2.00)^d^	21.00 (4.00)^e^

*Individual Mann–Whitney U tests were applied among all groups to assess differences between age (in years), levels of education (in years) and Mini Mental State Examination (MMSE) scores. Gender-ratio differences were calculated with a chi-square test.*

*^a^Significantly lower than middle-aged controls p < 0.05.*

*^b^Significantly lower than older controls p < 0.05.*

*^c^Significantly lower than all patient groups p < 0.05.*

*^d^Significantly lower than all control groups p < 0.05.*

*^e^Significantly lower than all other groups p < 0.05.*

### Neuropsychological Assessment

All participants completed an extensive neuropsychological test battery assessing a range of cognitive domains. The tests used in the present study were chosen to reflect test batteries routinely administered in tertiary-care clinics. This battery, in particular, has demonstrated high discriminatory power in differentiating patients with neurodegenerative conditions, even in early disease stages, from healthy groups, as well as patients with functional memory disorders (Venneri et al., 2011; Wakefield et al., 2014, 2018). A comprehensive list of included tests can be seen in Table 2 and detailed descriptions of these can be found in Neuropsychological Assessment, 5th Edition (Lezak et al., 2004). For between-group comparison of test performance, raw scores taken from healthy adults were converted to *z*-scores based on the mean and standard deviation of the overall healthy reference sample. In the case of patients, similarly standardization of scores was based on norms obtained from the same healthy reference sample. This harmonization served to standardize data variability according to each group’s age range and assess variability in test scores in relation to healthy functioning. Medians and interquartile ranges of the standardized test scores for each group can be found in Table 2.

**TABLE 2:** Median (and interquartile range) of cognitive test *z*-scores for each participant group, with results of a Kruskal–Wallis *H* test.

		Young (*n* = 75)	Middle aged (*n* = 70)	Older (*n* = 75)	aMCI (*n* = 75)	naMCI (*n* = 60)	Dementia (*n* = 60)	Kruskal–Wallis *H*	*df*	*p*-Value
Raven’s Progressive Matrices Z Scores	Median (IQR)	0.36 (1.03)	0.36 (1.12)	−0.24 (1.96)^ab^	−0.62 (1.80)^ab^	−0.86 (1.59)^ab^	−2.19 (2.55)^f^	135.628	5	<0.001
	Mean Rank	281.03	286.66	216.69	180.89	165.88	84.27			
Letter Fluency Z Scores	Median (IQR)	−0.15 (0.98)	0.29 (1.20)	−0.04 (1.33)	−0.59 (1.51)^b^	−0.92 (1.40)^abc^	−1.28 (1.11)^abcd^	80.104	5	<0.001
	Mean Rank	240.81	279.09	235.15	186.12	164.13	117.67			
Category Fluency Z Scores	Median (IQR)	−0.22 (1.28)	0.16 (1.07)	−0.21 (1.75)	−1.52 (1.08)^abc^	−1.08 (1.45)^abc^	−2.36 (1.10)^f^	198.682	5	<0.001
	Mean Rank	266.61	307.12	264.36	137.93	174.4	66.27			
Digit Cancelation Z Scores	Median (IQR)	0.53 (1.04)	0.40 (0.97)	−0.16 (1.62)^a^	−0.77 (1.76)^ab^	−1.09 (1.60)^abc^	−2.06 (2.65)^abcd^	139.272	5	<0.001
	Mean Rank	290.05	279.47	226.39	175.67	152.57	90.49			
Similarities Z Scores	Median (IQR)	−0.53 (1.29)^b^	0.32 (1.44)	0.32 (1.50)	−0.52 (1.65)^bc^	−1.00 (1.64) ^abc^	−1.98 (1.90) ^abcd^	118.952	5	<0.001
	Mean Rank	220.28	279.77	276.88	195.93	151.65	94.02			
Token Test Z Scores	Median (IQR)	0.19 (1.25)	0.73 (0.54)	0.19 (1.62)	−0.31 (2.25)^b^	−1.04 (2.54)^abc^	−2.63 (3.28)^f^	131.499	5	<0.001
	Mean Rank	257.77	286.02	237.98	201.64	147.64	81.59			
Rey-Osterrieth Complex Figure – Copy Z Scores	Median (IQR)	0.48 (0.76)	0.10 (1.29)	0.06 (1.46)^a^	−0.51 (1.94)^ab^	−0.81 (2.05)^ab^	−2.80 (4.32)^f^	108.129	5	<0.001
	Mean Rank	296.23	242.95	224.16	184.13	177.59	95.43			
Rey-Osterrieth Complex Figure – Recall Z Scores	Median (IQR)	0.65 (1.19)	−0.09 (1.19)	−0.30 (1.23^a^	−1.54 (1.33)^abc^	−0.89 (1.21)^ab^	−2.37 (0.73)^f^	191.69	5	<0.001
	Mean Rank	316.13	269.83	231.45	136.79	196.67	68.53			
Stroop Test – Time Interference Z Scores	Median (IQR)	0.50 (0.80)	0.10 (0.84)	−0.38 (1.53)^ab^	−0.88 (2.14)^ab^	−1.15 (1.98)^ab^	−1.26 (3.92)^ab^	87.65	5	<0.001
	Mean Rank	301.58	251.5	188.64	172.43	166.93	144.77			
Stroop Test – Error Interference Z Scores	Median (IQR)	0.23 (0)	0.23 (0.05)	0.28 (0.05)	−0.13 (2.08)^abc^	0.19 (2.49)^abc^	−4.98 (6.70)^f^	116.16	5	<0.001
	Mean Rank	238.92	273.84	270.27	174.59	171.9	92.27			
Digit Span Forward Z Scores	Median (IQR)	0.12 (1.00)	−0.11 (1.55)	−0.11 (2.10)	−0.20 (1.07)	−0.49 (1.36)	−1.07 (1.51)^abcd^	40.01	5	<0.001
	Mean Rank	254.13	233.52	213.01	202.99	195.18	131.68			
Digit Span Backward Z Scores	Median (IQR)	−0.29 (2.25)	−0.29 (0.93)	−0.29 (1.09)	−0.11 (1.23)	−0.83 (1.23)	−1.14 (0.72)^f^	55.16	5	<0.001
	Mean Rank	245.01	237.67	242.48	196	191.66	115.78			
Prose Memory Test – Immediate Recall Z Scores	Median (IQR)		0.02 (1.24)	−0.25 (1.36)	−22 (1.55)^bce^	−0.50 (1.28)	−2.33 (1.48)^f^	151.84	4	<0.001
	Mean Rank		242	216.46	128.01	193.7	57.43			
Prose Memory Test – Delayed Recall Z Scores	Median (IQR)		−0.06 (1.66)	−0.28 (1.50)	−1.84 (1.24)^bce^	−0.70 (1.97)^bc^	−2.66 (3.84)^f^	183.1	4	<0.001
	Mean Rank		248.77	237.2	117.54	175.45	56.09			
Verbal Paired Associates Learning Test Z Scores	Median (IQR)	0.42 (1.50)	−0.04 (1.34)	−0.50 (1.24)^a^	−1.06 (1.16)^abc^	−0.67 (1.19)^ab^	−2.09 (1.54)^f^	174.14	5	<0.001
	Mean Rank	303.03	272.09	231.76	153.5	193.52	63.99			
Confrontation Naming Test Z Scores	Median (IQR)	−0.02 (1.78)	0.64 (1.01)	−0.02 (1.56)	−0.19 (1.63)	−0.19 (1.63)	−1.42 (3.41)^f^	40.11	5	<0.001
	Mean Rank	230.97	252.21	207.92	203.51	209.83	127.9			

*Table shows results of a Kruskal–Wallis H test with post hoc Dunn tests and a Bonferroni correction for multiple comparisons (significance set at p < 0.05). Significant differences between groups are indicated as:*

*^a^Significantly lower than young controls p < 0.05.*

*^b^Significantly lower than middle aged controls p < 0.05.*

*^c^Significantly lower than older controls p < 0.05.*

*^d^Significantly lower than aMCI p < 0.05.*

*^e^Significantly lower than naMCI p < 0.05.*

*^f^Significantly lower than all other groups p < 0.05.*

### Network Formation

For network formation, standardized test scores for each of the healthy control groups were recalculated based on the mean and standard deviation of their own age group. For patient groups, the same standardized scores were used, as in the between-group comparisons, based on the means and standard deviations of matched controls. Although graphs representative of psychological profiles have been often constructed based on Gaussian Graphical Models, the major part of cognitive scores in our six groups was not normally distributed and, therefore, was unsuitable for this approach. Within-group test-to-test correlations were thus computed between standardized test scores of each of the 16 cognitive measures using a non-parametric version of the partial-correlation procedure, based on *Spearman’s rho*, controlling for age (in years) and level of education (in years). The covariates age and education were chosen to reflect two demographic factors that have been found to mediate cognitive functioning most strongly among healthy populations and as such are the basis of the majority of normative studies (Caffarra et al., 2002, 2003; Alviarez-Schulze et al., 2021; Hammers et al., 2021; Lee et al., 2021; Málišová et al., 2021). While many additional factors may have a modulatory role in cognition, the inclusion of these two variables alone allows for parsimony while still providing appropriate control over extraneous factors when assessing differences between clinical groups. In the youngest control group, the number of measures was reduced to 14 because the Prose Memory test was not part of the original testing protocol available for this age group. All cognitive test scores were adjusted (i.e., multiplying scores by −1 where needed) so that higher scores were always indicative of better performance. Correlation coefficients with a *p*-value less than 0.05 were considered significant, and those associated with a *p* < 0.1 and >0.01 were further tested with a permutation-based approach, using 5000 randomizations (Hayes, 1998). Absolute differences between original and resampling-based *p*-values were then inspected. No difference was larger than 0.01, indicating minimal effect of chance. From the correlation matrix, a binary adjacency matrix was then created for each group in which a one was given for a significant correlation and a zero for a non-significant correlation. As in previous work in this area, three negative correlation coefficients were removed at this point, one from each control group, in adherence with the validation of graph theory measures on positively connected networks (Kaiser, 2011; Kellermann et al., 2016). Negative correlations between tests of differing cognitive domains, in fact, have no clear theoretical explanation and may reflect more complex relationships than those represented by positive associations. These accounted for only 0.5% of the total number of correlations calculated across groups, and, as such, their impact on the graphs was assumed to be negligible. However, when applying bootstrapping these correlations retained their significance. To assess their influence on the network, therefore, an additional analysis was performed on the graphs of the healthy control groups including the three negative correlations.

For each group, a cognitive network was created that consisted of 16 nodes, representing each cognitive test (14 in the youngest control group), and a number of binary bidirectional links, or edges, between the nodes, representing significant positive correlations. Due to the use of the absolute threshold of *p* < 0.05, the number of edges differed between groups. A proportional threshold was avoided in this case due to the known potential of such thresholds to include spurious, non-significant correlation coefficients as edges (van den Heuvel et al., 2017). As one objective of the present study was to explore the concept of cognitive dedifferentiation in aging (Baltes et al., 1980; Baltes and Lindenberger, 1997), the use of an absolute threshold in this case was also considered more appropriate to highlight group differences in network density.

### Network Visualization

In order to visualize the structure of each cognitive network, the binary adjacency matrices for each group were exported to the Gephi software (Bastian et al., 2009), where the data were transformed into two-dimensional graphs. These were then displayed applying the Force Atlas algorithm (scaling = 1000, gravity = 100 with ‘prevent overlap’ selected) (ForceAtlas2, Jacomy et al., 2014). The algorithm forces poorly connected nodes apart while pulling well connected nodes together, improving the structural visualization of each graph.

To quantify network structure further, the community conformation of each graph was calculated with the Louvain community detection algorithm applied to each node using the Brain Connectivity Toolbox within MATLAB (Rubinov and Sporns, 2010). Nodes with high interconnectivity are grouped within modules, while nodes with low levels of connectivity are segregated from one another. This allows for the detection of sub-network communities that were color-coded accordingly in the graphs.

### Network Analysis

**FIGURE 1:**
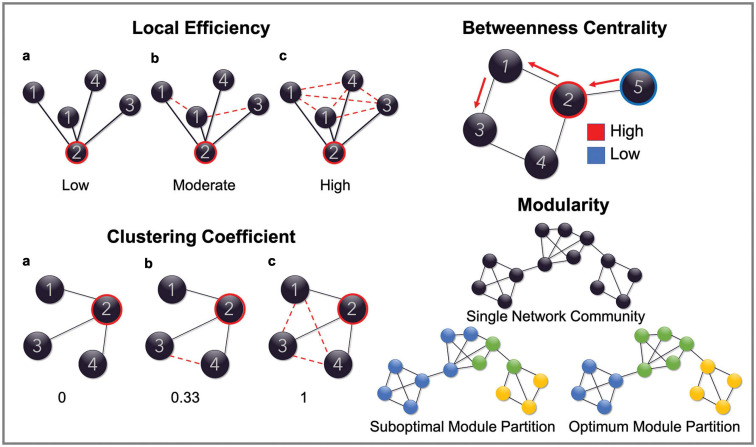
Schematic representation of network parameters. Figures are provided demonstrating the graph properties of local efficiency, clustering coefficient, betweenness centrality and modularity. Global efficiency is omitted as this measure reflects the same calculation as local efficiency but computed on the entire graph. In the top left, the local efficiency for node ‘2’ is low in example **a** because the average path length (path between any two nodes with the least number of edges) between its neighboring nodes is reasonably high (all are connected by two edges). It is high in example **c**, however, because the average path length is much lower (all nodes are connected by one edge), therefore demonstrating a highly efficient network structure. Clustering coefficient is a similar measure. In this case, node ‘2’, in the first instance, has a clustering coefficient of 0 because none of its neighboring nodes (nodes that are connected to it by an edge) are connected to each other by an edge. This increases to 0.33 in example **b**, because 1/3 of its neighboring nodes are connected and finally increases to 1 in example **c** because all of its neighboring nodes are now connected. In the top right of the figure, the betweenness centrality of node ‘2’ is high because it is included in the shortest paths between node ‘5’ and all the other nodes in the network. Node ‘5,’ however, has a low betweenness centrality as it is not included in the shortest paths between any of the other nodes. Finally, modularity is demonstrated in the bottom left by showing what constitutes an optimal modular structure.

Quantification of node-level network parameters was then performed on each adjacency matrix using the Brain Connectivity Toolbox run in a MATLAB environment. The specific parameters assessed included betweenness centrality, clustering coefficient and both the local and global efficiency of each individual node. Whole-network connection density was also computed for each graph. Together, these parameters function to quantify the integration and segregation of the network. Specifically, betweenness centrality represents a measure of how integral a node is to the efficient communication of the overall network and is calculated as the fraction of shortest paths (i.e., the path between any two given nodes that contains the least number of edges) between any two nodes that include the given node (Bullmore and Sporns, 2009; Rubinov and Sporns, 2010). Clustering coefficient is a measure of network segregation that assesses the interconnectivity of the nodes neighboring the node of interest. It is calculated as the fraction of a node’s neighbors (i.e., other nodes it is connected to by an edge) that are also connected to each other. Local efficiency is another measure of network segregation that is highly related to the clustering coefficient. It is calculated as the inverse of the average path length (i.e., the average length of the shortest paths between two nodes) between the neighbors of a given node. Global efficiency, on the other hand, is a measure of network integration. It is calculated as the inverse of the average path length between a given node and any other node in the network. Efficiency is an inverse measure because the shorter the shortest path length between nodes, the more efficient the connection between them. Figure 1 illustrates, using examples, how each of these parameters are measured. Connection density, sometimes referred to as *wiring cost*, simply refers to the fraction of edges that are present in the graph in relation to the number of possible edges that may be available, given the number of nodes. In the present study connection density was calculated on a node-by-node basis as the fraction of edges connected to a given node, out of the possible number of edges available if it were connected to all other nodes in the network. Please refer to Bullmore and Sporns (2009) for a succinct description of network parameters and Rubinov and Sporns (2010) for an overview of the mathematical formulas used to calculate the network metrics included in this study.

Further assessment of how network parameters differed between cognitive domains was conducted through the use of mean network metrics derived from select nodes. The values of network metrics derived from sub-sets of nodes, chosen according to their cognitive domain, were averaged to give a mean value for each metric for four different cognitive domains. Tests reflective of memory function, abstract reasoning, semantic processing, and executive functioning were grouped, in the first instance, on a theoretical basis so that metrics relating to memory function were calculated using a mean score derived for each group from the nodes corresponding to recall of the Rey-Osterrieth Complex Figure, both Prose Memory measures and the Verbal Paired Associates Learning Test of the Wechsler Memory Scale (Wechsler, 1997). Mean metrics for semantic processing were calculated using data derived from the Similarities sub-set of the Wechsler Adult Intelligence Scale (Wechsler, 2008), the Category Fluency Test and the Confrontation Naming Test. Similarly, mean abstract reasoning metrics were calculated again using the Similarities and Category Fluency Test but including Raven’s Progressive Matrices in place of the Confrontation Naming Test. Finally, metrics relating to executive functioning were calculated using data derived from each of the Stroop Test interference measures, the Letter Fluency Test and the backward version of the Digit Span Test. The intra and inter-domain consistency of performance on cognitive tests corresponding to each domain was further assessed using test-to-test correlations calculated for the whole cohort, and comparing the coefficients of correlation calculated between the tests in each domain group (i.e., the six coefficients of correlation calculated between four tests of memory) with the coefficients of correlation calculated between every single test not included in the given domain (i.e., the 48 coefficients of correlation calculated between each test of memory and every single non-memory test). This was done correcting for age, education and, as this analysis was done using the entire cohort, MMSE scores. After transforming the correlation coefficients into z scores, the results indicated extremely robust consistency within the memory domain (intra-domain coefficient: 0.392; inter-domain: 0.142) and a positive trend within the semantic domain (intra-domain coefficient: 0.278; inter-domain: 0.222) and within the abstract-reasoning domain (intra-domain coefficient: 0.332; inter-domain: 0.246). The executive domain showed instead comparable levels of inter- and intra-domain consistency without any visible trend. This is in line with a multi-componential view of executive functioning, described by recent models not as a unitary function, but as a set of distinct and only partially correlated functions (Miyake et al., 2000; Packwood et al., 2011).

### Statistical Analyses

According to a Shapiro–Wilk test, continuous demographic data were largely non-normally distributed. As such, between-group differences were assessed using individual two-tailed Mann–Whitney *U* tests. Differences in categorical variables were assessed using chi-square tests (Table 1). Neuropsychological test data were also largely non normally distributed and were, therefore, assessed using the non parametric Kruskal–Wallis *H* test with *post hoc* Dunn tests and a Bonferroni correction for multiple comparisons, with significance set at *p* < 0.05 (Table 2).

Between-group differences in network parameters were assessed using individual two-tailed Mann–Whitney *U* tests with significance determined as *p* < 0.05. A Bonferroni correction for multiple comparisons was further applied.

## Results

### Cognitive Task Performance

The results of a Kruskal–Wallis *H* test comparing task performances between groups are reported in Table 2. Patient groups demonstrated significant differences from healthy controls in a number of cognitive tests aligned to the severity and sub-type of their diagnosis (see Table 2).

Older adults performed significantly worse than both younger groups on Raven’s Progressive Matrices and took significantly more time than both when completing the Stroop Test. Furthermore, older adults performed significantly worse than the youngest group on tasks including Digit Cancelation, copying and recall of the Rey-Osterrieth Complex Figure and the Verbal Paired Associates Learning Test.

No significant differences were found between the older control group and younger groups on tasks relating to language or semantic memory. The middle-aged group, however, performed significantly better than younger controls on the Similarities task.

### Visualization of Network Structure

**FIGURE 2:**
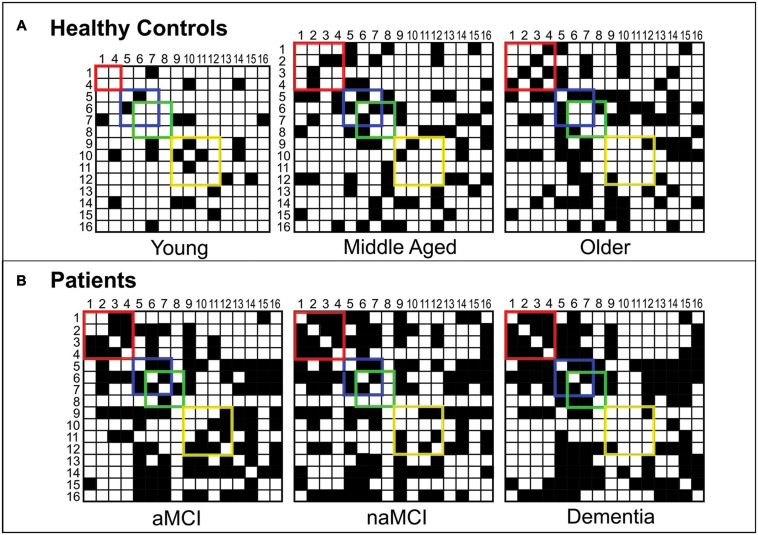
Binary adjacency matrices for participant groups. Panel **(A)** shows the binary adjacency matrices of healthy controls (Young, Middle Aged and Older groups). Panel **(B)** shows the binary adjacency matrices of patients (aMCI, naMCI and dementia groups). A grid square filled in black represents a significant positive correlation (an edge) between two cognitive tests (nodes). Correlations between memory tests are enclosed by the red square, abstract reasoning by the blue square, semantic processing by the green square and executive functions by the yellow square. For ease, cognitive tests have been converted to numbers so that: 1 = Rey-Osterrieth Complex Figure – Recall, 2 = Prose Memory Test – Immediate Recall, 3 = Prose Memory Test – Delayed Recall, 4 = Verbal Paired Associates Learning Test (WMS), 5 = Raven’s Progressive Matrices, 6 = Similarities (WAIS), 7 = Category Fluency Test, 8 = Confrontation Naming Test, 9 = Letter Fluency Test, 10 = Stroop Test – Time Interference, 11 = Stroop Test – Error Interference, 12 = Digit Span Test – Backward, 13 = Digit Span Test – Forward, 14 = Digit Cancelation Test, 15 = Rey-Osterrieth Complex Figure – Copy, 16 = Token Test.

Figure 2 shows the binary adjacency matrices created for each of the six participant groups.

**FIGURE 3:**
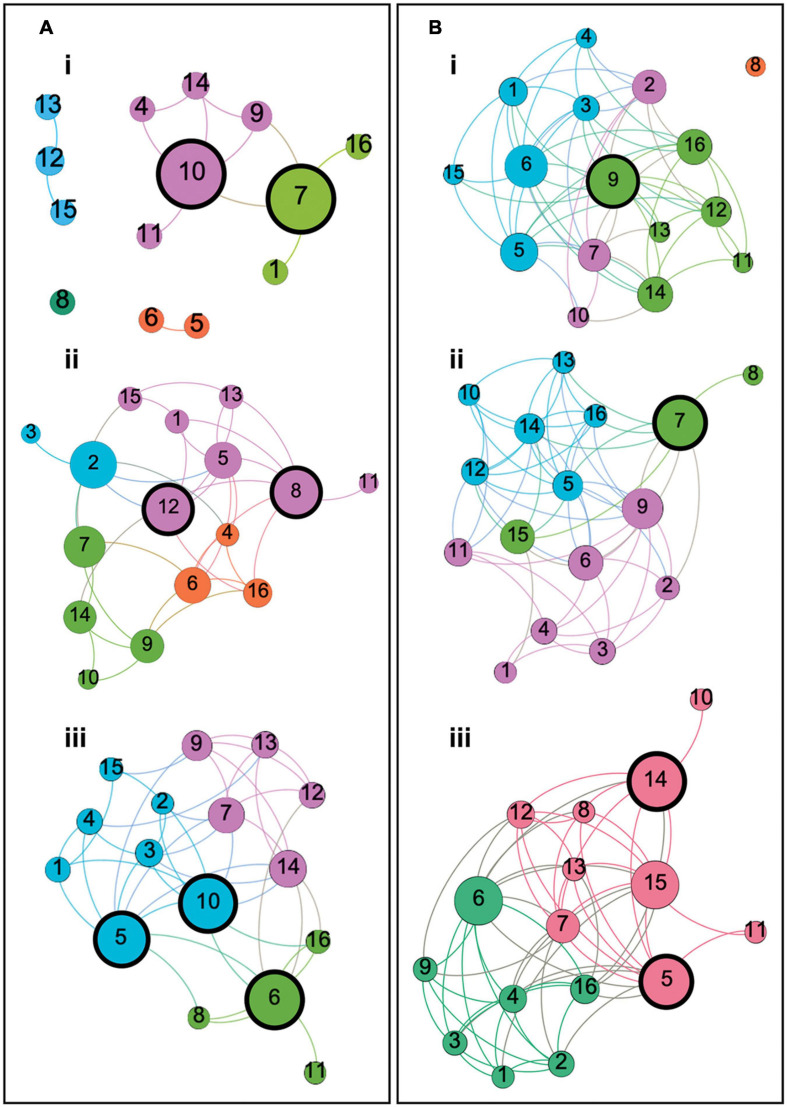
Two dimensional graphs representing the structure of each participant group’s cognitive network. Panels **(A)** represent the graphs of Young, Middle Aged and Older controls, respectively. Panels **(B)** represent the graphs of naMCI, aMCI and AD dementia groups, respectively. Each node corresponds to a cognitive test and each edge represents a significant correlation between tests. Color is reflective of modularity class, identified using the Louvain community detection algorithm, and node size is representative of betweenness centrality relative to the individual graph. Modules in each group loosely correspond to cognitive domains described as follows: **(Ai)** Young controls: Four modules (and one disconnected node) with two modules corresponding to memory and language function (green) and memory and executive function (pink). **(Aii)** Middle-aged controls: Four modules corresponding to language comprehension and semantic processing (orange), verbal memory and executive functioning (green), recollective memory function (blue) and a less clearly defined module corresponding to multiple domains including memory, language functioning and abstract reasoning (pink). **(Aiii)** Older controls: Three modules corresponding to episodic memory and visuoconstructive ability (blue), language comprehension and semantic processing (green) and verbal memory and executive functioning (pink). **(Bi)** naMCI patients: Three heterogeneous modules (and one disconnected node) corresponding to episodic memory, abstract reasoning and visuoconstructive ability (blue), verbal memory and executive function (pink) and attention, executive function, verbal memory and language comprehension (green). **(Bii)** aMCI patients: Three modules corresponding to language and memory function (pink), semantic processing and visuoconstructive ability (green) and a heterogeneous module including tests of language comprehension, abstract reasoning, verbal memory and executive function (blue). **(Biii)** Dementia patients: Two modules with one consisting only of tests of language and memory function (green) and another consisting of tests corresponding to any other cognitive domain (red). Nodes are labeled as: 1 = Rey-Osterrieth Complex Figure – Recall, 2 = Prose Memory Test – Immediate Recall, 3 = Prose Memory Test – Delayed Recall, 4 = Verbal Paired Associates Learning Test (WMS), 5 = Raven’s Progressive Matrices, 6 = Similarities (WAIS), 7 = Category Fluency Test, 8 = Confrontation Naming Test, 9 = Letter Fluency Test, 10 = Stroop Test – Time Interference, 11 = Stroop Test – Error Interference, 12 = Digit Span Test – Backward, 13 = Digit Span Test – Forward, 14 = Digit Cancelation Test, 15 = Rey-Osterrieth Complex Figure – Copy, 16 = Token Test.

Notable qualitative differences were apparent between groups in terms of network organization. Two-dimensional representations of each graph can be seen in Figure 3. Among healthy control groups there were substantial differences in community structure. The younger group presented with a sparse network, including many nodes with no network connections, whereas both older groups showed more interconnected networks with definable community structures. Where modularity calculated in the middle-aged group revealed four sub-network communities, in the older group this was reduced to three. In healthy older and middle-aged adults, sub-network modules could be loosely described as corresponding to cognitive domains, as described in Figure 3. In younger controls, however, although some evidence of domain specificity was present, module formation was limited due to the sparsity of the graph.

Network modules defined within the naMCI group were highly heterogeneous and adhered less clearly to cognitive domains, particularly when compared with the other two patient groups. In this case, tests of language, memory and semantic processing were relatively evenly spread across three sub-network modules, whereas, among aMCI and dementia patients, module class was heavily influenced by a node’s relation to memory functioning. The aMCI group similarly demonstrated three discernible sub-network communities. Unlike the naMCI group, however, nodes relating to language and semantic processing within the aMCI network were less evenly spread between modules. aMCI patients instead presented with one well-defined module for memory function and two less distinct modules, including a very large module comprised of nodes relating to multiple domains and a very small three-node module including two tests of semantic function and one unrelated task of visuoconstructive ability. In this sense, the cognitive network of the aMCI group was more similar to the dementia patients, in which only two modules were present. In this case, sub-networks were clearly delineated into one module consisting only of tests of language and memory function and another consisting of tests corresponding to any other cognitive domain. In both groups, the module relating to memory function included the same six cognitive tests.

Overall, there was distinctly less differentiation between cognitive domains among healthy older individuals when compared with the younger groups. This was clearly exacerbated in disease groups, particularly among the aMCI and dementia patients.

The size of nodes shown in the graphs in Figure 3 reflects their respective betweenness centrality. Nodes with a high betweenness centrality can be described as demonstrating hub-like properties (van den Heuvel and Sporns, 2013). Network hubs with a betweenness centrality greater than 1.5 standard deviations above the mean for each group are highlighted with thick black borders in Figure 3.

Since network structure can be influenced by both age and education, *post hoc* analyses were carried out including and excluding education as a regressor in the analytic procedures. Across groups, the total number of correlations that were altered by the presence or absence of education as a covariate in the analysis was 39 out of the 603 coefficients of correlation (6.5%) and this appeared particularly relevant for tests of semantic processing/abstract reasoning and memory.

### Network Metrics

**FIGURE 4:**
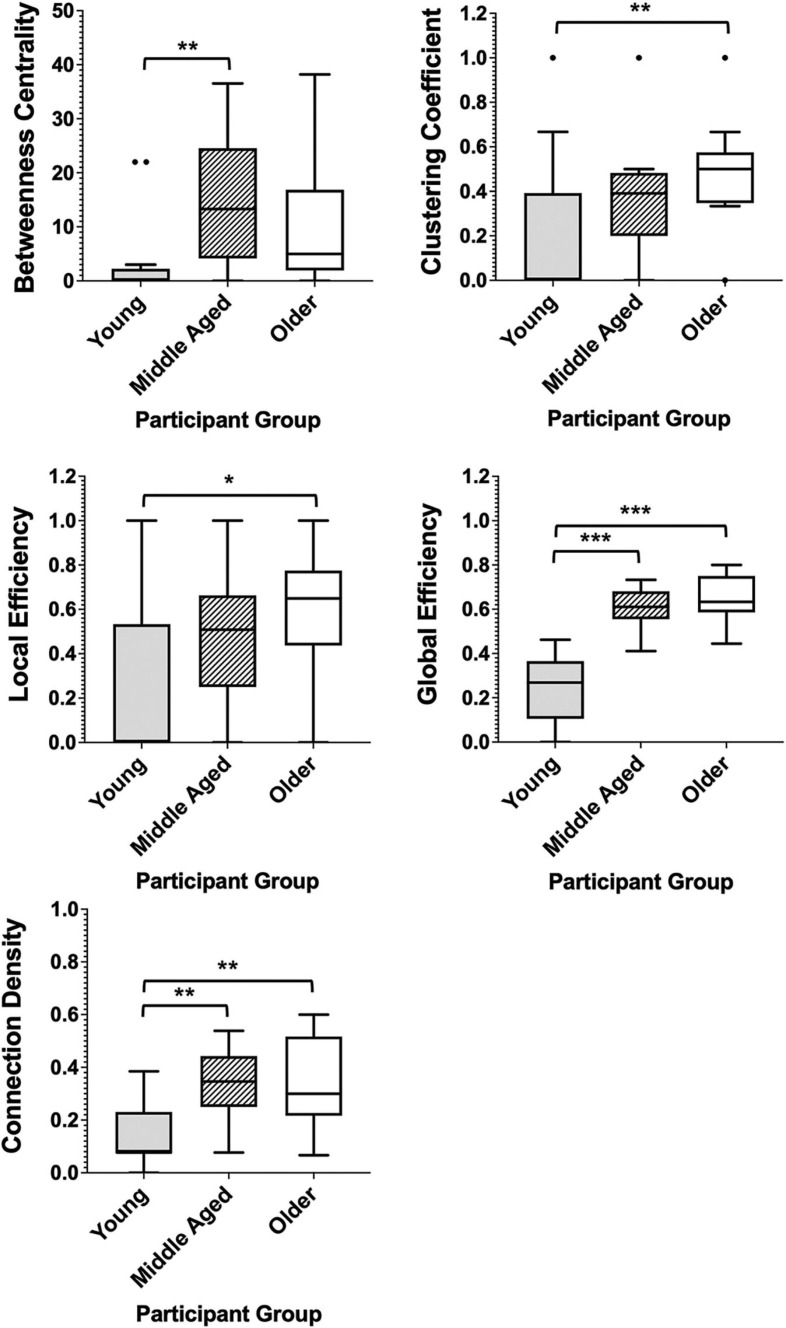
Box plots showing the median and interquartile range of network metrics for the graphs of the healthy groups. Significant differences calculated using independent two-tailed Mann–Whitney *U* tests. *p < 0.05, **p < 0.01, ***p < 0.001 (Bonferroni correction for multiple comparisons).

Among healthy adults, measures of both network segregation and network integration showed linear differences across groups with average global and local efficiency measures, clustering coefficients and connection density being lowest in the youngest group and highest in the older control group. Betweenness centrality, however, demonstrated a different pattern. This measure was lowest among the youngest controls and highest in the middle-aged group, with older controls showing a decrease in this measure compared with middle aged adults. Significant differences, calculated by Mann–Whitney *U* tests, can be seen in Figure 4.

The analysis on the graphs of the healthy groups was repeated including the three negative correlations. The findings confirmed that the inclusion of these negative correlations had very little impact on the results (see Supplementary Material for a full description of the findings and Supplementary Figure 1 for their graphical representation).

**FIGURE 5:**
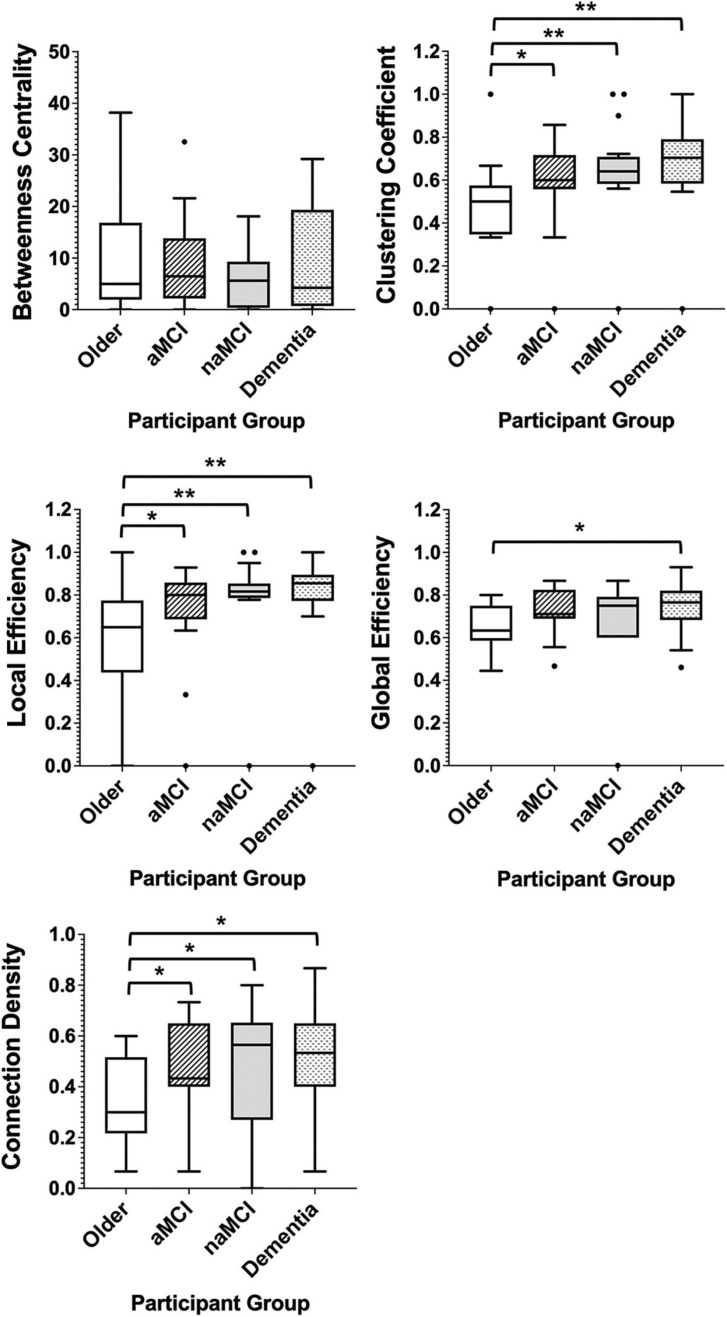
Box plots showing the median and interquartile range of network metrics for the graphs of the older controls and patient groups. Significant differences calculated using independent two-tailed Mann–Whitney *U* tests. *p < 0.05, **p < 0.01.

In patient groups, the measures of network segregation, clustering coefficient and local efficiency, demonstrated the greatest differences when compared with controls, with all three groups demonstrating significantly greater levels of each measure when compared with healthy older adults (Figure 5). Network integration also differed between patients and healthy adults, with all patient groups also demonstrating significantly higher connection densities than the healthy older group and dementia patients further having significantly higher levels of global efficiency than the control group (Figure 5). Comparison of all six groups can be found in Supplementary Figure 2, demonstrating the exacerbation of age-related changes among patients.

### Cognitive Domains

Qualitative differences between groups in neuropsychological profiles, relating to the network metrics of four cognitive domains, are detailed below.

**FIGURE 6:**
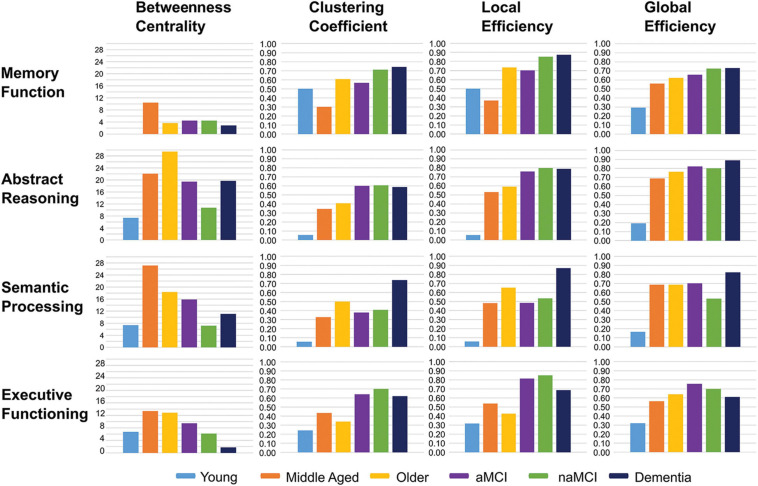
Bar charts showing the average network metrics for each cognitive domain across participant groups.

As with the network average, global efficiencies in all cognitive domains showed a linear trend of differences between control groups, with the youngest group having the lowest efficiency scores and the oldest group the highest (Figure 6). In general, global efficiencies relating to all domains were higher in aMCI patients than controls and highest in dementia patients, with the exception of executive functioning.

Local efficiency and clustering coefficient tended to be higher within patient groups, across domains, compared with controls, particularly in executive functioning and abstract reasoning. In the dementia group this was again the case in semantic processing and memory domains (Figure 6).

Among control groups, local efficiency and clustering coefficient ranged from lowest in the youngest group and highest in the older group in both abstract reasoning and semantic processing. In the case of memory, however, the middle-aged group had the lowest measures of both parameters, and the highest in executive functioning, compared with the other healthy groups (Figure 6).

Betweenness centrality was lowest among the youngest controls in all cognitive domains, aside from executive function, where the dementia group presented with a lower average, and highest among the middle-aged group in all domains with the exception of abstract reasoning in which it was highest among older healthy adults.

All patient groups showed lower average betweenness centralities than older controls in the domain of semantic processing and considerably lower centralities in abstract reasoning. This was also true in the domain of executive functioning. In contrast, average betweenness centralities in the memory domain were very similar between patient groups and healthy older adults. Both patients and healthy older adults, however, demonstrated substantially lower betweenness centralities in this domain when compared with the middle-aged group (Figure 6).

Finally, we examined in more detail the profile of multi-domain aMCI patients (*n* = 64 of the original 75). The resulting graphs were very similar to those of the original aMCI group.

## Discussion

### Network Alterations in Healthy Aging

In the healthy groups, significant differences in network connection density between the youngest adults and both older groups, demonstrate definable differences between the stages of healthy aging in the independent function of separate cognitive domains. Although not a significant difference, greater connection density was also apparent among the oldest control group when compared with the middle-aged group. Such findings provide supporting evidence for the existence of age-related dedifferentiation between cognitive domains, particularly in the transition between early life (<40 years old) and middle age, a change that may occur in a similar manner to the age-related dedifferentiation of the neural response (Koen and Rugg, 2019).

A further indication of age-related cognitive dedifferentiation is reflected by the results relating to modularity. As in previous studies reporting similar age-related decreases in network modularity within brain systems (Cao et al., 2014; Chan et al., 2014; Han et al., 2018; Chong et al., 2019), the number of modules delineated in the graphs of the control groups was lowest among older adults and highest among younger adults, where a sparsely connected graph resulted in a number of disconnected modules. Given that the nature of module identification serves to maximize the number of within-module edges while minimizing the number of between-module edges (Rubinov and Sporns, 2010), a low number of definable modules can indicate a globally well-connected graph with relatively low distinction between separable node groups of higher interrelatedness. However, it must be noted that an absence of connections between nodes may also result in a graph with low modularity. To some extent, the calculation of community modules is comparable to factor analysis, reducing a larger number of variables to a smaller number of influential factors that, in the present study, may be best described in terms of cognitive domain (Park et al., 2012; Agelink van Rentergem et al., 2020). A previous study by Li et al. (2004), which used a principal component analyses of 15 cognitive tests, conducted on six age groups from childhood to old age, revealed fewer dominant principal components in childhood, late adulthood and old age than in adolescence to middle age; a finding that the authors attributed to the *differentiation–dedifferentiation hypothesis* of intellectual ability across the lifespan (Reinert, 1970; Baltes et al., 1980; Baltes and Lindenberger, 1997). In addition to the *dedifferentiation hypothesis* of aging, the *differentiation hypothesis* of human intelligence, originally introduced by Garrett (1946), posits that in early development general childhood cognitive ability is gradually broken down into the distinct domain-specific functioning that characterizes adult cognition. As such, the results presented by Li et al. (2004), as well as those of the present study, are in line with a theory of cognition that suggests that the interrelatedness of our cognitive domains, highlighted here by the topological parameters of cognitive networks, is a dynamic process, heavily affected by the processes of maturation and senescence.

Unlike factor analysis, however, modularity, in this case, is calculated as part of a wider, complex set of metrics and therefore functions as a contributing factor to the thorough description of the entire cognitive scaffold. Graph theory parameters assessing both the segregation and integration of the overall network, also showed significant differences between the stages of healthy aging. Differences tended to present in a linear manner, with older adults demonstrating significantly higher levels of both efficiency measures and clustering coefficients than the youngest controls and the middle-aged group having averages that were intermediate between the two. As measures of network segregation, both differences in clustering coefficient and local efficiency indicate a difference in the local interconnectivity between neighboring nodes, suggesting higher levels of cognitive network segregation in older adults. Conversely, as a measure of network integration, significantly higher levels of global efficiency among both the older groups, when compared with young controls, is an indication of greater network-wide interconnectivity (Rubinov and Sporns, 2010). A network that demonstrates high levels of network segregation, in combination with high levels of network integration, can be described as presenting with the property of ‘small-worldness’ (Watts and Strogatz, 1998). Increasing small-worldness across the stages of healthy aging may again reflect a shift in cognitive functioning from the domain-related independent functioning of early and middle adulthood to a more generalized network-dependent functioning in old age (Reinert, 1970; Baltes et al., 1980; Baltes and Lindenberger, 1997). Findings relating to betweenness centrality among the control groups may be a further reflection of such a shift. Significantly lower levels of betweenness centrality in the younger group compared with the middle-aged group in this study is likely to be largely reflective of the limited edges present in the graph of this group and the disconnection of network modules, resulting in a lack of overall betweenness centrality. Such a finding, although largely explained as a product of differing connection densities, is still in line with the concept that in younger age our cognitive abilities are largely dissociable from one another, with less influence from general factors dictating performance of multiple tasks. Significantly greater betweenness centrality in middle age, however, may reflect the emergence of a greater reliance on a reduced number of cognitive factors, as per the findings of factor analysis studies (Li et al., 2004), to facilitate multiple disparate cognitive functions. Contrastingly, however, the oldest group of healthy controls presented with a lower, although not significantly, average betweenness centrality than the middle-aged group. Although not a direct measure of interconnectivity, betweenness centrality may be influenced by the density of the network. In accordance with this, higher connection densities and lower betweenness centralities were also apparent among patient groups when compared with controls. As a fractional value, dependent on the number of shortest paths between any two nodes to which a given node belongs, lower betweenness centrality values may be influenced by the average shortest path length of the graph. A graph containing a higher number of binary, undirected edges will inherently present with a shorter average path length, as was demonstrated by the linear increase in connection density and global efficiency outlined between participant groups. This, therefore, may reduce the fraction of shortest paths to which a given node belongs, thereby reducing that node’s betweenness centrality (see Rubinov and Sporns, 2010 for the relative arithmetical formula). It could be argued, therefore, that a potential limitation of this study was the use of an absolute threshold to identify graph edges, leading to differences in network density between the groups. As outlined in the methods, however, an absolute threshold was chosen to avoid the inclusion of non-significant correlations in the networks (van den Heuvel et al., 2017), particularly where significant correlations were highly sparse, as in the youngest control group. Use of an absolute threshold, in this case, instead provided an arguably more accurate visualization of how the structure of cognitive networks differs between the stages of aging, with network density itself proving an integral alteration that likely reflects an age-related breakdown of highly segregated domain-specific functioning.

Despite the controversies surrounding the *differentiation–dedifferentiation hypothesis* (de Frias et al., 2007; Tucker-Drob, 2009; Tucker-Drob et al., 2019), the use of graph theory provides additional evidence for age-related alterations in the topology of the cognitive network itself, in a step away from the relatively simplified operationalization of cognitive dedifferentiation put forward by previous factor analyses. Graph theory metrics not only allow for similar exploration of cognitive dedifferentiation, via connection density and modularity, but further establish age-dependent differences in our cognitive system between the stages of aging from a relatively disparate conformation of independent domains to a well-established network, that, in accordance with brain networks, may be defined by the property of small-worldness and the presence of a number of influential hubs (Bullmore and Sporns, 2009) that underlie general cognitive ability.

Across individual domains, the linear pattern of differences in clustering coefficient, as well as in global and local efficiency, was relatively well preserved among the three healthy groups and the levels of each parameter were relatively similar for each domain within groups. Betweenness centrality of individual domains, however, revealed notable differences depending on age group. Young adults demonstrated similarly low levels of betweenness centrality across domains whereas middle aged and older adults showed a much greater variance in betweenness centrality, with both groups showing the highest levels of this measure in the domains of abstract reasoning and semantic processing. In particular, when compared with the middle-aged group, the oldest group of healthy adults demonstrated much higher levels of betweenness centrality in the domain of abstract reasoning, while showing much lower levels in the memory domain. Such findings may reflect changes in the reliance on differing domain-specific functions across the lifespan. High levels of betweenness centrality in the domains of semantic processing and abstract reasoning, in both the middle-aged and older groups, support the hypothesis that there is an increased reliance on this type of crystallized intelligence, which is developed throughout the lifetime (Cattell, 1971), to support healthy cognitive function, across multiple domains, as we age and our fluid abilities decline (Harada et al., 2013). Such an interpretation has previously been put forward by factor analysis studies that have demonstrated a greater correspondence between crystallized and fluid abilities in older age (Li et al., 2004), and more recently, neuroimaging studies have even outlined age-related neural network alterations as a possible mechanism for this phenomenon (Spreng et al., 2018). This is, therefore, in accordance both with the neuropsychological findings of the present study that showed no age-related declines in semantic memory function among healthy groups, instead demonstrating a significantly better performance among middle-aged adults compared with the younger group on the Similarities task, as well as with previous studies demonstrating the relative maintenance of semantic processing and language functions in normal aging, despite notable declines in other areas such as episodic memory and processing speed (Nyberg et al., 1996; Salthouse, 2009, 2019). Low levels of memory-related betweenness centrality in the older healthy group, when compared with the middle-aged group, therefore, may reflect the tendency for this cognitive function to decline in normal aging (Harada et al., 2013) and, as such, play a diminishing role in the connectivity of the wider cognitive network.

### Network Alterations in Disease

Significant differences in cognitive network topology were further established between healthy groups and those with pathological cognitive impairment. Firstly, all patient groups presented with significantly greater levels of connection density than healthy adults, a finding reflected by previous network analyses of cognitive profiles in healthy aging and Alzheimer’s disease (Tosi et al., 2020). Higher connection densities among disease populations, in comparison with controls, may reflect a greater tendency for individuals in these cohorts to demonstrate a concordance in their dysfunction across a range of disparate cognitive tests. Despite the propensity for neurodegeneration to affect certain domains more than others, a level of global decline is often apparent in the presence of disease (Amieva et al., 2005, 2008; Bäckman et al., 2005; Grober et al., 2008). In accordance with the suggestion that age-related cognitive dedifferentiation may reflect the effects of undiagnosed disease processes (Batterham et al., 2011), it is likely that the impact of neuropathological damage on overall effortful cognitive processing may result in shared variances in task performance across a number of domains and therefore an exacerbated expression of dedifferentiation in the cognitive network. Such findings, therefore, support the use of graph theory measures to highlight system-wide alterations in cognitive functioning among disease populations (Garcia-Ramos et al., 2016; Kellermann et al., 2016). Cognitive covariance networks such as this may, furthermore, provide a means to partial out the effect of global declines when assessing neuropsychological functioning among cognitively impaired patient groups. The suggestion that performances on individual cognitive tests are influenced not only by deficits in a specific domain but furthermore, by some general disease-related constraint on cognition, may be somewhat problematic in the pursuit of early differential diagnosis of neurodegenerative etiologies. The quantification of cognitive profiles, using graph theoretical techniques, eliminates the potential for global impairments to impact on the identification of disease-specific cognitive change. By evaluating the topology of the cognitive network and the inter-relatedness of its various domains, the underlying neuropsychological mechanisms influencing individual task performances can be more readily examined. As such, this technique may provide a clearer indication of the specific alterations driving cognitive impairments and, therefore, be beneficial for differential diagnosis.

Regarding modularity, of particular interest was the finding that the discernible modules in the graphs of the aMCI and dementia groups were clearly separated by their relation to language and memory function, a characteristic identified in Alzheimer’s disease in the study by Tosi et al. (2020) and in both aMCI and Alzheimer’s dementia patients in the study by Ferguson (2021) that was not apparent among the healthy older participants or the naMCI patients. Given the corroboration of this finding by previous network analyses (Tosi et al., 2020; Ferguson, 2021), and the fact that both these domains are known to be significantly affected in Alzheimer’s disease (McKhann et al., 2011), the graphs presented here can be said to reflect accurate neuropsychological profiles that are characteristic of this type of neurodegeneration. Furthermore, in line with the findings of Ferguson (2021), similarities between the graph of the aMCI and Alzheimer’s dementia groups demonstrate that this pattern may be discernible even in the early stages of disease, being clearly distinguishable from the profile of healthy older individuals. In an extension of Ferguson’s findings, the lack of similarity between the networks of the Alzheimer’s dementia group and the naMCI patients, who may be more likely to represent the prodromal stages of a differing neurodegenerative condition (Petersen et al., 2001; Petersen, 2004; Busse et al., 2006; Petersen and Negash, 2008; Ferman et al., 2013), suggests that cognitive network analysis may be a useful technique for characterizing differential cognitive profiles between disparate neurological populations (Garcia-Ramos et al., 2016; Kellermann et al., 2016; Jonker et al., 2019; Tosi et al., 2020) not only in the dementia stages of disease but, furthermore, in the prodromal stages. Future work in this area will endeavor to include a wider range of etiologies to determine applicability of these methods in the distinction of divergent presentations of cognitive decline.

Network segregation appeared significantly affected by the presence of disease, with both local efficiency and clustering coefficients being significantly higher among patient groups when compared with healthy older controls. Furthermore, all patient groups demonstrated significantly higher connection densities when compared with controls and dementia patients further presented with significantly higher global efficiencies, suggesting an alteration to network integration. Previously, neuroimaging studies assessing the topology of both structural and functional neural networks have demonstrated significant alterations in the small-world properties of such networks in Alzheimer’s disease patients, at varying disease stages, including patients with prodromal and even preclinical manifestations of disease (He et al., 2008; Yao et al., 2010; Zhao et al., 2012; Tijms et al., 2013a, 2016; Wang et al., 2013; Zhou and Lui, 2013; Brier et al., 2014; Pereira et al., 2016, 2018; Dai et al., 2019; Franciotti et al., 2019). Though such alterations tend to indicate a breakdown in the small-worldness of brain networks among patients with Alzheimer’s disease, the results presented here, in clustering and efficiency measures, suggest that, at the cognitive level, functional domains may show greater levels of network integration and segregation, in terms of their statistical correlation, among patient groups, compared with controls. Given that this is one of the first investigations using graph theoretical methods to model neuropsychological profiles within a cognitively impaired, neurodegenerative population, it remains unclear what the relationship may be between underlying alterations of physiological network topology and the differences seen at a cognitive level. However, in line with the findings relating to connection density, and the demonstration by previous studies of a significant relationship between brain network graph theory parameters and measures of cognition (Shu et al., 2012; Tijms et al., 2014, 2018; Dicks et al., 2018; Verfaillie et al., 2018), the results presented here could be explained as a reflection of the influence of significant changes in brain network functioning on the global cognitive system that may influence the variance in task performance in a similar manner across domains, resulting in greater interconnectivity among clusters and across the wider network.

Average network parameters for particular cognitive domains allowed for more nuanced insight into domain-specific differences between the groups. In general, network parameters in each domain revealed a similar pattern of differences between patients and controls as findings relating to the overall graph, with measures of clustering coefficient, local efficiency and global efficiency tending to be lowest in young healthy adults and highest in patients. However, measures of betweenness centrality indicated some notable differences between patients and controls in how integral differing domains were to the efficient communication of the network. In particular, betweenness centralities in the domains of abstract reasoning and semantic processing were distinctly lower among patient groups when compared with healthy adults, with the relative levels of this metric in abstract reasoning being the greatest difference seen between MCI patients and healthy older adults. Contrastingly, betweenness centralities in the domain of memory were very similar between older healthy adults and the patient groups, where both had distinctly lower averages when compared with middle-aged adults. A diminished role of memory function in the cognitive network, reflected here by low betweenness centralities, therefore, appears to be a property of both healthy and pathological aging, likely resultant of declines in episodic memory, that are a well-established characteristic of both the healthy aging process (Nyberg et al., 1996; Rönnlund et al., 2005) and of Alzheimer’s-related cognitive impairment (Dubois et al., 2007; McKhann et al., 2011). Similarities in network parameters relating to memory function between healthy adults and those with a cognitive impairment, emphasize the inadequacy of episodic memory measures alone to characterize pathological decline in age-related neurodegenerative disease, especially in its early stage.

Low levels of betweenness centrality in semantic and abstract reasoning domains among the patient groups, when compared with controls, however, may reflect a disease-related decrease in the reliance on such functions to facilitate cognition. This finding is supported by studies showing that significant declines in semantic processing tend to occur in concomitance with the earliest pathological stages of Alzheimer’s disease (Joubert et al., 2010, 2020; Barbeau et al., 2012; Venneri et al., 2019; Vonk et al., 2020) and, therefore, demonstrates the possibility for graph theoretical methods to highlight differences in neuropsychological profiles in normal aging and disease, particularly through the assertion that age-related reliance on crystallized semantic ability, or knowledge, to facilitate cognitive function is greatly diminished in the presence of pathology. Evidence from longitudinal studies has previously implicated semantic memory impairment as one of the earliest markers of cognitive decline in individuals who go on to develop Alzheimer’s disease dementia (Amieva et al., 2008; Vonk et al., 2020) and, as such, neuropsychological profiling in this manner may have significant implications in the identification of incipient disease processes.

### Limitations

A possible limitation of this study was the exclusion of extraneous factors, such as education and imaging features from the analytical process of cognitive network formation. In a *post hoc* analysis, education was excluded from the set of regressors in order to compare education-corrected and education-uncorrected coefficients of correlation. Except for the youngest controls, education had some influence over the significance of correlation coefficients seen between cognitive tests. As such, the number and conformation of edges that are moderated by education attainment differ across diagnoses, and appear to be particularly relevant among the middle aged and naMCI groups. The percentage of coefficient of correlations altered by inclusion/exclusion of education, was however small. These findings, therefore, suggest that education does indeed play a role in the formation of cognitive networks and suggests that cognitive reserve, a concept heavily linked to educational attainment (Stern, 2009; Meng and D’Arcy, 2012), may be partially reflected in the strength of the relationship between differing cognitive domains and their shared influence in the completion of cognitive tasks.

A second potential limitation might be related to lack of inclusion of imaging features in the analysis. While the inclusion of neuroimaging is certainly important, the current literature already includes many studies that have investigated the utility of graph theoretical methods in assessing alterations in neural networks at varying stages of Alzheimer’s disease (He et al., 2008; Yao et al., 2010; Zhao et al., 2012; Tijms et al., 2013a, 2016; Wang et al., 2013; Zhou and Lui, 2013; Brier et al., 2014; Pereira et al., 2016, 2018; Dai et al., 2019; Franciotti et al., 2019). While previous studies have successfully demonstrated an association between brain network topology and cognitive function (Shu et al., 2012; Tijms et al., 2013b, 2014, 2018; Dicks et al., 2018; Verfaillie et al., 2018) researchers have yet to investigate the potential parallels between neural network dysfunction and topological alterations in cognitive systems. Potential future research, therefore, may take the direction of mixed MRI/cognitive graphs that may be better equipped to identify clinical profiles with greater specificity.

## Conclusion

In conclusion, the present study outlines a new approach to the modeling of age- and disease-related cognitive decline. Quantification of the structure of cognitive networks within both patients and healthy controls revealed compelling evidence for the existence of measurable alterations in neuropsychological profiles throughout healthy aging that are distinct from alterations associated with underlying pathological change. Furthermore, topological distinctions between the cognitive networks of differing diagnostic groups suggests some potential for the further exploitation of graph theory methods for the differentiation of cognitive profiles associated with varying disease etiologies. Finally, network parameters in specific cognitive domains highlighted a prominent role of crystallized abilities in the network connectivity of healthy older adults that appeared greatly diminished among patient groups, a defining feature that may serve to inform future investigations into novel diagnostic approaches to pathological cognitive impairment.

## Data Availability Statement

The raw data supporting the conclusions of this article will be made available by the authors, without undue reservation.

## Ethics Statement

The studies involving human participants were reviewed and approved by the West of Scotland Regional Ethics Committee 5, Ref. No.: 19/WS/0177. The patients/participants provided their written informed consent to participate in this study.

## Author Contributions

AV and MDM conceived this study and contributed to study design. AV contributed to data collection. LW contributed to study design, carried out data analysis, and drafted this manuscript. MDM and AV provided critical review of the manuscript. AV finalized this manuscript. All authors approved the final version of this manuscript.

## Disclaimer

The views expressed are those of the authors and not necessarily those of the NHS, the NIHR or the Department of Health.

## Conflict of Interest

The authors declare that the research was conducted in the absence of any commercial or financial relationships that could be construed as a potential conflict of interest. The handling editor declared a past collaboration with one of the authors AV.
