# Supplementary material for: A Graph Theory Approach to Clarifying Aging and Disease Related Changes in Cognitive Networks
Source: Front Aging Neurosci. 2021 Jul 12;13:676618. doi: 10.3389/fnagi.2021.676618 (PMC8311855; doi:10.3389/fnagi.2021.676618)
Supplement: Supplementary file 1 [file Data_Sheet_1.pdf]

Supplementary material

Additional network metrics analysis including negative correlations

The negative correlations that were found in this study were as follows: In the youngest control group, the number of errors made on the Stroop Task was negatively correlated with the Similarities subset of the WAIS (in this study Stroop Error scores had been adjusted so that higher scores reflected less errors. i.e., an error of 1 was recorded as -1), similarly in both the middle aged and older control groups negative correlations were found between the number of errors on the Stroop Task and the Verbal Paired Associates Learning Test of the Wechsler Memory Scale. To assess the influence of these correlations on the network, analysis was performed on the graphs of the healthy control groups including these negative correlations. The inclusion of these had very little impact on the results (see Supplementary Fig 1.).

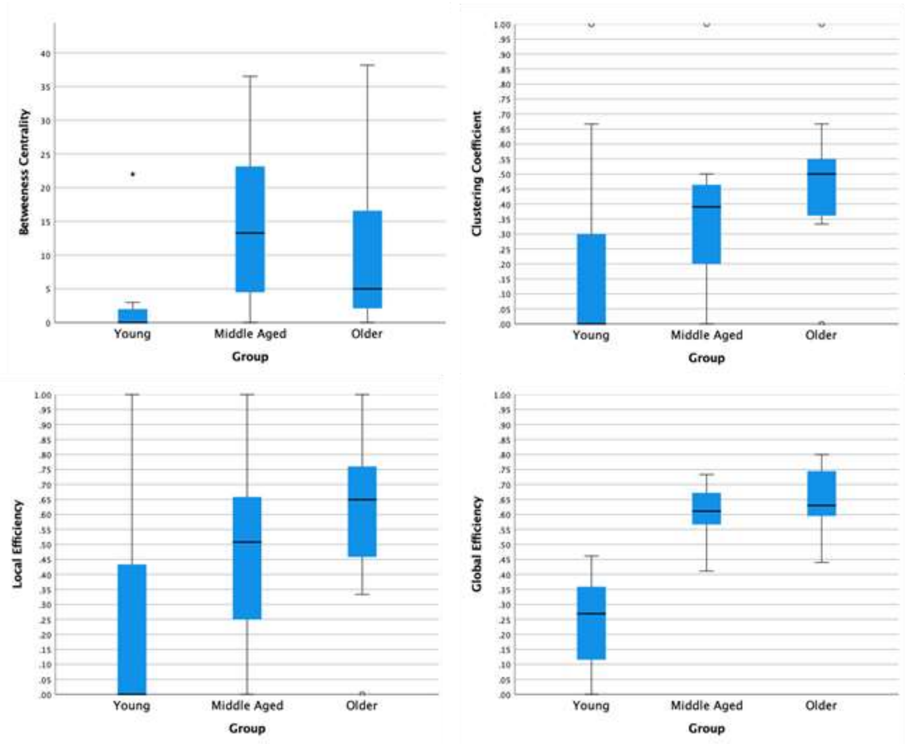

Original Results

(a)

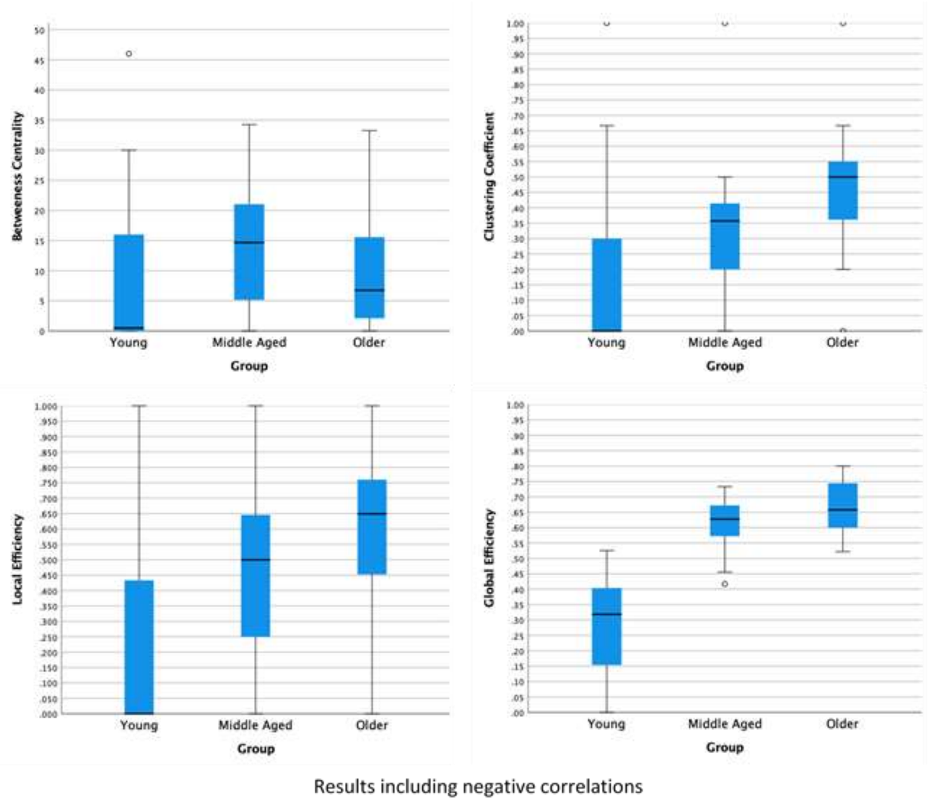

(b)

<u>Supplementary Figure 1</u> Boxplots showing the median and range of network parameters in each of the control groups with (a) and without (b) the inclusion of negative correlations.

A substantial difference was apparent however, within the youngest group in the measure of betweenness centrality, due to the addition of an edge between the Similarities and Stroop Task Error nodes which resulted in a connection between a cluster of two nodes to the largest cluster in the graph that had previously been disconnected. The increase in betweenness centrality in this group therefore eliminated the significant difference originally found between the young and middle-aged groups on this measure (see **Fig. 4**). Another small alteration was the difference between the middle aged and older groups on the measure of clustering coefficient, which was previously approaching significance (p = .07), was found to reach the level of significance when including the negative correlations (p = .047).

Supplementary Figure 2.

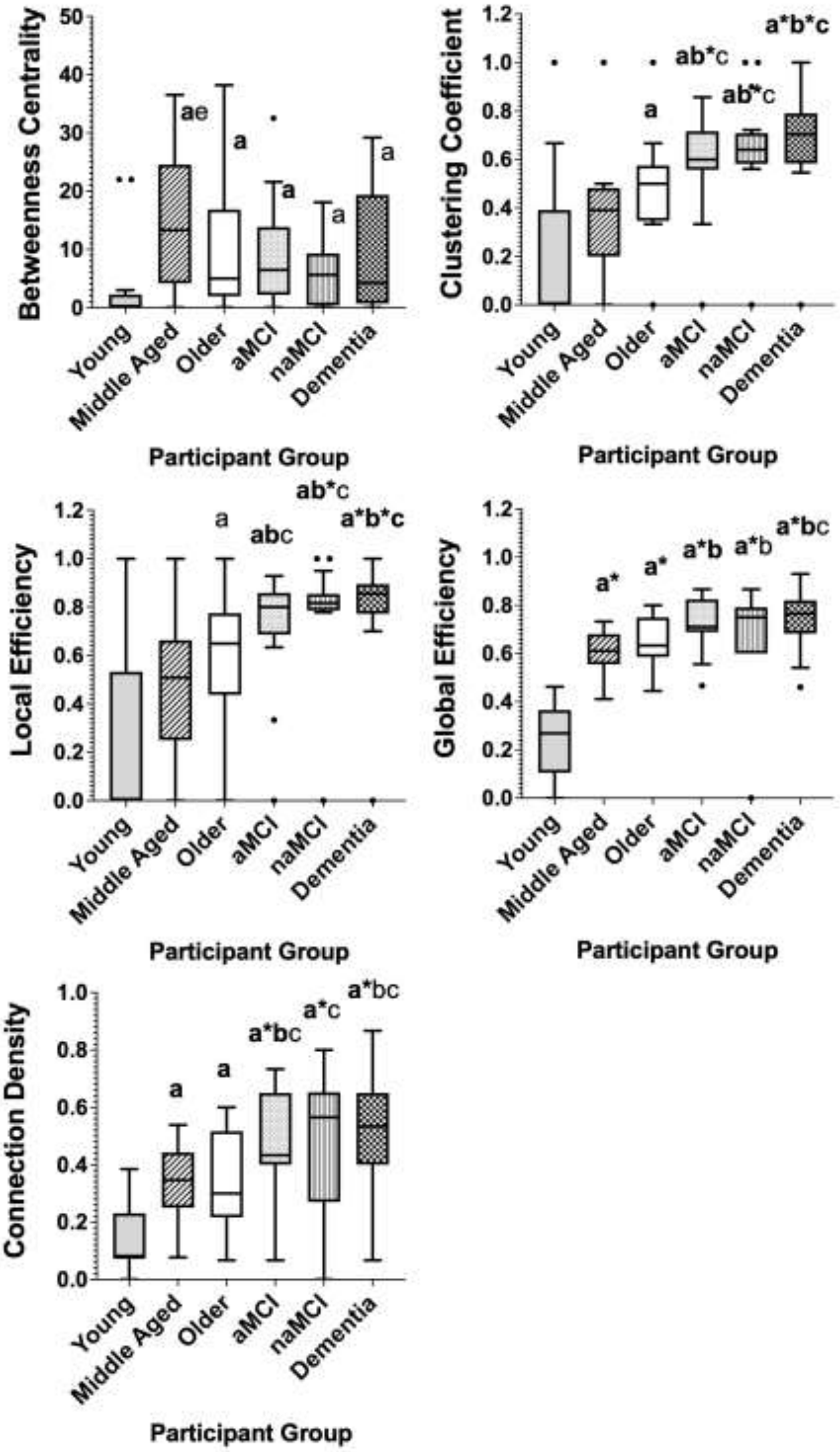

<u>Supplementary Figure 2.</u> **Box plots showing the median and interquartile range of network metrics for the graphs of each group.** Significant differences ($p < .05$) calculated using independent two-tailed Mann-Whitney U tests.

a Significantly greater than young controls.

b Significantly greater than middle aged controls.

c Significantly greater than older controls.

d Significantly greater than aMCI.

e Significantly greater than naMCI.

Bold letters indicate $p < .01$.

*significant with Bonferroni correction for multiple comparisons ($p < .001$).
